# An Unusually Short Latent Period of Therapy-Related Myeloid Neoplasm Harboring a Rare MLL-EP300 Rearrangement: Case Report and Literature Review

**DOI:** 10.1155/2019/4532434

**Published:** 2019-10-02

**Authors:** Reina Takeda, Kazuaki Yokoyama, Seiichiro Kobayashi, Toyotaka Kawamata, Sousuke Nakamura, Tomofusa Fukuyama, Mika Ito, Nozomi Yusa, Eigo Shimizu, Nobuhiro Ohno, Rui Yamaguchi, Seiya Imoto, Satoru Miyano, Kaoru Uchimaru, Arinobu Tojo

**Affiliations:** ^1^Department of Hematology/Oncology, Research Hospital, The Institute of Medical Science, The University of Tokyo, Tokyo 108-8639, Japan; ^2^Division of Cellular Therapy, The Institute of Medical Science, The University of Tokyo, Tokyo 108-8639, Japan; ^3^Division of Molecular Therapy, The Institute of Medical Science, The University of Tokyo, Tokyo 108-8639, Japan; ^4^Department of Applied Genomics, Research Hospital, Institute of Medical Science, The University of Tokyo, Tokyo 108-8639, Japan; ^5^Laboratory of DNA Information Analysis, Human Genome Center, Institute of Medical Science, The University of Tokyo, Tokyo 108-8639, Japan; ^6^Division of Health Medical Data Science, Health Intelligence Center, Institute of Medical Science, The University of Tokyo, Tokyo 108-8639, Japan; ^7^Laboratory of Tumor Cell Biology, Department of Computational Biology and Medical Science, Graduate School of Frontier Sciences, The University of Tokyo, Tokyo 108-8639, Japan

## Abstract

Therapy-related myeloid neoplasm (t-MN) is a late and lethal complication induced by chemotherapy and/or radiation therapy. Hematological malignancy is one of the most common primary diseases in patients with t-MN. However, the occurrence of t-MN in adult T-cell leukemia/lymphoma (ATL) patients is rarely reported, possibly due to the dismal prognosis of ATL per se. Here, we report a 62-year-old female who developed t-MN only three months after the completion of conventional chemotherapy and anti-CCR4 antibody for ATL acute type. The patient presented with persistent fever and monocytosis without any evidence of infectious diseases. Bone marrow examinations revealed chronic myelomonocytic leukemia-like disease with a chromosomal translocation of t(11;22)(q23;q13) as a solo cytogenetic abnormality, resulting in the diagnosis of t-MN. Next-generation sequencing analysis identified a rare chimeric transcript, MLL-EP300, without any additional somatic mutations. Although the patient underwent allogenic hematopoietic stem cell transplantation, she died of viral encephalomyelitis at 7 months after diagnosis of t-MN. Since recent therapeutic advances have extended the survival of patients with ATL, further evaluation of the long-term risks of developing t-MN in these patients is warranted.

## 1. Introduction

Therapy-related myeloid neoplasm (t-MN) is a late complication induced by chemotherapy and/or radiation therapy for both malignant diseases and nonmalignant diseases [1]. Typically, t-MN has a latency period of at least a few years following exposure to therapeutic agents [2]. Median survival time after diagnosis of t-MN is 8 months, and five-year overall survival is less than 10% [2]. As well as breast cancer, hematological malignancy including non-Hodgkin's lymphoma, Hodgkin's lymphoma, and multiple myeloma is observed as the most common primary disease in patients with t-MN [1–3]. However, there have been only a few reports on t-MN developing in adult T-cell leukemia/lymphoma (ATL) patients [4–8]. The incidence risk of t-MN in ATL patients was estimated as 2.1% [4], which is much lower than that of non-Hodgkin's lymphoma (5–10%) [3, 9]. This is possibly due to the dismal survival outcomes of ATL per se [4, 5]. In particular, the acute type of ATL has a median survival time of only 8.3 months despite intensive therapies [10]. Thus, patients with ATL rarely survive long enough to develop secondary malignancies. Recent introduction of an anti-CCR4 antibody called mogamulizumab has improved prognosis in ATL patients [11, 12]. Here, we report a case of t-MN developing shortly after intensive chemotherapy combined with a humanized anti-CCR4 antibody, mogamulizumab, for ATL, along with the results of molecular investigation using next-generation sequencing and literature review.

## 2. Case Presentation

**Figure 1:**
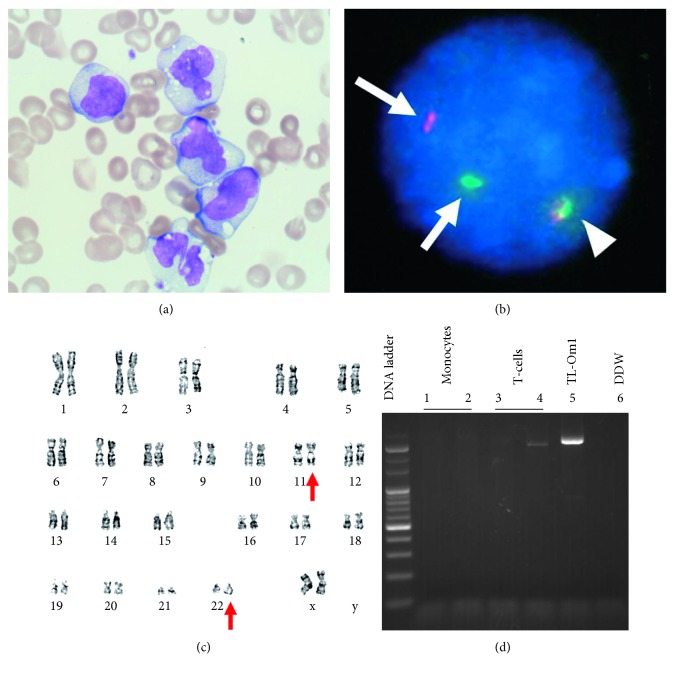
Characteristics of MLL-EP300-positive therapy-related myeloid neoplasm in the present case. (a) Bone marrow (BM) showed numerous differentiated monocytes without any increase in the number of myeloblasts or adult T-cell lymphoma/leukemia (ATL) cells (May–Giemsa stain, original magnification, 1,000x). Some monocytes exhibited slightly atypical nuclei. (b) Fluorescence in situ hybridization (FISH) analysis for gene rearrangements involving in the mixed lineage leukemia (MLL) gene at 11q23 was performed. The normal MLL gene exhibited a yellow signal (arrow head), whereas a split MLL gene exhibited as a pair of green and red signals (arrows). The BM sample from the present case showed that 78.5% of the interphase cells were positive for the split MLL gene signals. (c) G-banding of the BM material revealed 46,XX, t(11;22)(q23;q13)[17]/46,XX[3]. Red arrows indicate the abnormal chromosomes involving the translocation. (d) Human T-cell leukemia virus type 1 (HTLV-1) provirus DNA analysis was performed by using inverse polymerase chain reaction. CD14-sorted monocytes (lanes 1, 2) showed no bands. CD3-sorted T-cells (lanes 3, 4) failed to show a visible band with the reproducibility, indicating that the case was negative for a major ATL clone. TL-Om1 was an HTLV-1-infected cell line as positive control (lane 5), and distilled and deionized water (DDW) was used for negative control (lane 6).

A 62-year-old female patient with the acute type of ATL received five sessions of mLSG-15 therapy combined with mogamulizumab [12]. A dose-intensified chemotherapy called mLSG-15 therapy is commonly used as an initial treatment for aggressive ATL and consists of VCAP (vincristine, cyclophosphamide, doxorubicin, and prednisone), AMP (doxorubicin, ranimustine, and prednisone), and VECP (vindesine, etoposide, carboplatin, and prednisone) [12]. She achieved a complete response (CR) from ATL. Three months later, she was referred to our hospital for allogeneic hematopoietic stem cell transplantation (alloHSCT). On admission, she had persistent fever accompanied by repetitive skin rash and arthralgia. The skin rash exhibited patches of 2-3 cm in diameter and sometimes harbored a subcutaneous mass with pain. Interestingly, these skin and joint symptoms always recovered spontaneously in a few days before recurring in different parts of the body. Peripheral blood (PB) examination showed WBC 4.9 × 10^9^/L (neutrophils 32.5%, lymphocytes 15.0%, monocytes 48.5%, myeloblasts 0.0%, abnormal lymphocytes 0.5%, and monocytoid cells 3.5%), Hb 9.6 g/dL, and platelets 87 × 10^9^/L. The absolute monocyte count in PB was 2.4 × 10^9^/L. Monocytosis had been persistently observed, although ATL cells had hardly been detected in PB by either morphological or immunophenotypic analysis. Lactate dehydrogenase increased slightly (LDH: 247 IU/L, normal range 105–211 IU/L). C-reactive protein was highly elevated (CRP: 20.46 mg/dL). The proviral load (PVL) of HTLV-1 was only 0.45%. A systemic computed tomography scan indicated no hepatosplenomegaly or lymphadenopathy or other signs associated with malignant diseases, infectious diseases, or inflammatory diseases. Bone marrow (BM) examination revealed a slight hypocellularity with a predominance of differentiated monocytes (58% of nuclear cell count) without an increase of blast cells (2%) (Figure 1(a)). Screening analysis for the representative 11 leukemic chimera genes including BCR-ABL1 by polymerase chain reaction was negative. Flow cytometric analysis revealed that the monocytes increased in the BM were positive for CD45, CD33, CD4, CD14, and HLA-DR but negative for CD2, CD13, and CD56. Fluorescence in situ hybridization (FISH) analysis showed split mixed lineage leukemia (MLL) gene signals in 78% of the interphase cells (Figure 1(b)) but no rearrangement signals with regard to two genes: platelet-derived growth factor receptor alpha and beta. Karyotyping analysis using the G-band method detected the t(11;22)(q23;q13) translocation as a solo cytogenetic abnormality (Figure 1(c)). This chromosomal aberration was not observed at the onset of ATL. Human T-cell leukemia virus type 1 (HTLV-1) provirus DNA analysis (via inverse polymerase chain reaction) of CD14-sorted monocytes revealed no monoclonal integration (Figure 1(d)) [13].

Therefore, the patient was diagnosed with therapy-related myeloid neoplasm (t-MN), which phenotypically resembled chronic myelomonocytic leukemia (CMML)-1. The latent period between the initial therapy and the onset of t-MN was 10 months. On the contrary, ATL was confirmed as CR, in accordance with the response criteria for ATL [14]. Our case did not have clinical evidence of ATL disease such as increase of ATL cells in PB and BM, swollen lymph nodes, hepatosplenomegaly, and skin involvement of ATL cells. Additionally, the results from PVL and inverse PCR analysis were also consistent with the conclusion that her ATL had remained in CR (Figure 1(d)). She underwent alloHSCT but died of viral complications of encephalomyelitis at 4 months after transplantation (7 months after diagnosis of t-MN).

In order to identify the partner gene of MLL gene rearrangement in this case, we performed RNA sequencing analysis. Total RNA was extracted from the patient's BM sample using a QIAGEN RNeasy Mini Kit (QUIAGEN, Venlo, Netherlands). cDNA libraries for next-generation sequencing were constructed from 24 ng of total RNA using a Truseq RNA Access Library Prep Kit (Illumina, San Diego, CA, USA). Each paired-end indexed library was sequenced to a length of 75 nucleotides per mate (2 × 75 bp) on a Nextseq instrument (Illumina). Sequence reads were processed by our in-house Genomon-RNA pipeline [15, 16] (available at http://genomon.readthedocs.org/ja/latest/, http://genomon.hgc.jp/rna/). Fusion transcripts were detected by Genomon-fusion. The Integrative Genomics Viewer (IGV) version 2.3.57 (https://software.broadinstitute.org/software/igv/download) was used to visualize the fusion-sequence. For validation of the fusion transcripts, reverse transcription polymerase chain reaction (RT-PCR) and Sanger sequencing were performed. Total RNA was extracted from the patient's BM sample using a PAXgene blood RNA kit (QIAGEN) and QIAqube. The RNA was reverse transcribed into cDNA by using SuperScript™ IV VILO™ Master Mix (Thermo Fisher Scientific, Massachusetts, USA). DNA was amplified and sequenced using Platinum Taq HF (Thermo Fisher Scientific) and the following primers: primer F 5′-GTGTGGGAGATGGGAGGCT-3′ from MLL exon 10 and primer R 5′-CCTCCATCTTCACTTCCTGGG-3′ from EP300 exon 15.

Next, to explore the molecular mechanisms of leukemogenesis in this case, we performed a targeted deep sequencing analysis. Flow cytometric sorting of the CD14-positive monocytes and CD4-positive T-cells (control 1) from the patient's peripheral blood was performed using a FACS Aria II (BD Biosciences, San Jose, CA). Oral epithelial cells (control 2) were collected via buccal swab from the patient. Targeted sequencing was performed using 20 ng of DNA via the TruSight Myeloid Panel on the MiSeq platform (Illumina), which included the analysis of the following 54 genes related to myeloid malignancies: ABL1, ASXL1, ATRX, BCOR, BCORL1, BRAF, CALR, CBL, CBLB, CBLC, CDKN2A, CEBPA, CSF3R, CUX1, DNMT3A, ETV6/TEL, EZH2, FBXW7, FLT3, GATA1, GATA2, GNAS, HRAS, IDH1, IDH2, IKZF1, JAK2, JAK3, KDM6A, KIT, KRAS, MLL, MPL, MYD88, NOTCH1, NPM1, NRAS, PDGFRA, PHF6, PTEN, PTPN11, RAD21, RUNX1, SETBP1, SF3B1, SMC1A, SMC3, SRSF2, STAG2, TET2, TP53, U2AF1, WT1, and ZRSR2. T cells and oral epithelial cells served as a germline control in this case. Bioinformatic analysis was performed using standard procedures [17].

**Figure 2:**
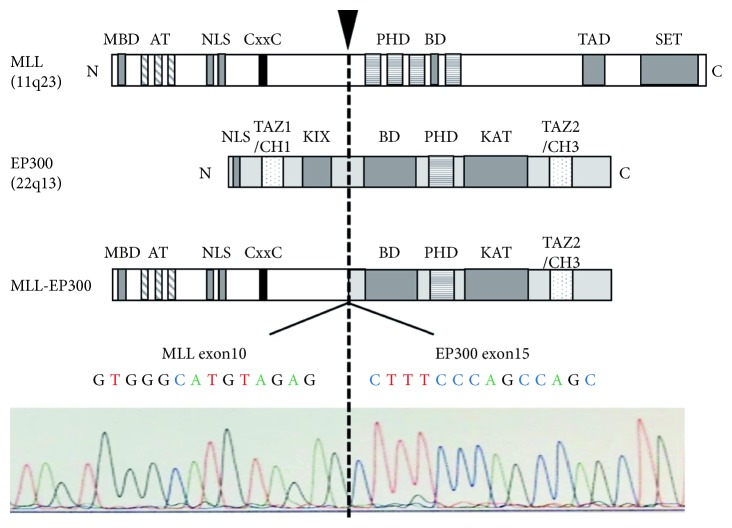
The structures of MLL, EP300, and MLL-EP300 fusion protein and the sequence of the fusion gene. RNA sequencing and RT-PCR followed by Sanger sequencing demonstrated that MLL exon 10 was fused in-frame to EP300 exon 15. The breakpoint is indicated by the arrow and dashed line. MBD, menin-binding domain; AT, AT hooks; NLS, nuclear localization signal; CxxC, motif recognizing unmethylated CpG dinucleotides; PHD, plant homeodomain fingers; BD, bromodomain; TAD, transactivation domain; SET, H3K4 histone methyltransferase domain; TAZ, transcriptional-adaptor zinc-finger domain; CH, cysteine/histidine-rich regions; KIX, kinase-inducible domain of the CREB-interacting domain; KAT, lysine acetyltransferase domain.

Cytogenetic and FISH analyses revealed that MLL on chromosome 11q23 was involved in gene rearrangement as the result of chromosomal translocation t(11;22)(q23;q13). RNA sequencing analysis followed by RT-PCR validation was performed to identify the partner gene of MLL rearrangement. RNA sequencing analysis detected a chimeric gene, MLL-EP300, formed by the in-frame fusion of MLL exon 10 to EP300 exon 15. The fusion gene was also confirmed by RT-PCR and Sanger sequencing (Figure 2) [18–20].

To seek the molecular details underlying leukemogenesis in this case, mutation screening analysis with a targeted deep sequencing method was performed. The panel was focused on myelodysplastic syndrome- (MDS-) and acute myeloid leukemia- (AML-) related genes. Among these 54 genes, SRSF2, TET2 and ASXL1 are frequently mutated in CMML patients [21]. However, any additional somatic mutations were not detected in our case (data not shown).

This study was approved by the Institutional Review Board of the Institute of Medical Science, the University of Tokyo, and informed consent was obtained from the patient in accordance with the Declaration of Helsinki.

## 3. Discussion

t-MN typically occurs as a late complication of chemotherapy and/or radiation administered for primary malignant diseases [1]. The most common subtype of t-MN is caused by alkylating agents and/or radiation after a long latency period of 5–7 years [1, 2]. This subtype is associated with the type of MDS that often progresses to AML and is frequently characterized by complete or partial deletion of chromosomes 5 and 7 [1, 2]. In contrast, the second major subtype caused by topoisomerase II inhibitors presents as overt AML with a shorter latent period of 2–3 years [1, 2, 22]. This topoisomerase II inhibitor-associated t-MN is associated with balanced translocation, such as chromosomal translocations involving the MLL at 11q23; the runt-related transcription factor 1 (RUNX1) at 21q22; and a promyelocytic leukemia-retinoic acid receptor alpha (PML-RARA) fusion resulting from the t(15; 17) [1, 2, 22]. Our present case had a therapeutic history of topoisomerase II inhibitors including etoposide and exhibited a balanced chromosomal translocation t(11;22)(q23;q13) resulting in MLL gene rearrangement. Collectively, these clinical features suggest that our case was consistent with t-MN induced by topoisomerase II inhibitors. Of note, our case had a shorter latency period of 10 months from the diagnosis of ATL.

The rare chimera MLL-EP300 has been reported in three different cases so far (Table 1) [16–18]. In all four cases including our case, MLL-EP300-positive leukemia emerged as t-MN following a medical history of malignancies. Although all the primary malignancies were treated with topoisomerase II inhibitors, other clinical features were quite varied. For example, there were variations in diagnostic ages, types of primary malignancies, latent periods, and locations of fusion breakpoints [18–20]. Intriguingly, only our case exhibited a phenotype of sustained monocytosis mimicking CMML-like disease, whereas the other three were acute myeloid leukemia (AML). In addition, the former three cases had one or more additional cytogenetic abnormalities other than t(11;22)(q23;q13), but only our case showed t(11;22)(q23;q13) as a solo cytogenic abnormality and had no additional cytogenetic abnormalities. Because only a limited number of cases were observed, it remains unclear whether and how differences of chromosomal breakpoints can affect leukemogenesis and the clinical features in MLL-EP300-positive leukemia. Molecular profiling by next-generation sequencing analysis is valuable to understand the pathogenesis and invent better therapeutic strategies. The genetic investigation with targeted deep sequencing demonstrated that our case had no additional somatic mutations, strongly implying that MLL-EP300 was a principal driver gene causing t-MN in this case.

EP300 is a rare fusion partner of MLL rearrangements among various translocations involving in MLL gene identified [18–20]. Interestingly, each MLL rearrangement can have different phenotypes and mechanisms for leukemogenesis, depending on the fusion partners [23]. Somatic mutations of EP300 gene have been found in hematological malignancies including MDS [24]. A recent study showed that loss of function of EP300 accelerates leukemic transformation of MDS cells [25]. On the contrary, it was reported that lysine acetyltransferase (KAT) activity of EP300 promotes leukemogenesis in AML by acetylating H3K18 [26]. In addition, a study showed that bromodomain of EP300 regulates functions of myeloid-derived suppressor cells via controlling H3K27 acetylation [27]. As shown in Figure 2, MLL-EP300 fusion protein in this case includes both KAT domain and bromodomain of EP300, indicating that this fusion protein could induce myeloid transformation via altering epigenetic modifications in hematopoietic cells. Functional analysis of MLL-EP300 fusion protein is necessary to elucidate leukemogenesis induced by this rare translocation.

In previous studies, t-MN developing after ATL had a devastating prognosis, regardless of primary ATL disease status [4–8]. Most of the patients died only a few months after diagnosis of t-MN [4–8]. Various cytogenetic abnormalities not limited to MLL rearrangements were detected in t-MN after ATL [4–8]. The risk of t-MN among ATL patients was estimated as 2.1% [4]. This is obviously lower than the incidence rate of t-MN in patients with non-Hodgkin's lymphoma (estimated as 5–10%) [1, 3, 9]. This is possibly due to the dismal survival outcomes of ATL per se [4, 5]. However, it is important to note that these previous studies on t-MN after ATL were published before the introduction of mogamulizumab, which has recently been reported to improve the prognosis of patients with aggressive ATL [11, 12]. Given such recent therapeutic progress, further evaluation of the long-term risks of t-MN in ATL patients should be warranted.

In conclusion, we present a case of t-MN, with a rare MLL-EP300 rearrangement and an unusually short latent period. Additionally, according to the previous literature of t-MN with MLL-EP300 rearrangement, only our case exhibited a phenotype of CMML. Based on the cytogenetic abnormality of balanced translocation, a topoisomerase II inhibitor was thought to be the causative factor for t-MN in this case. Because survival time increases for patients with ATL who had prior exposure to chemotherapeutic agents, the clinician must keep in mind the cumulative risk of t-MN in these patients as well.

## Figures and Tables

**Table 1:** A summary of cases of therapy-related MLL-EP300-positive leukemia (cases 1–4).

Case	Age/sex	Phenotype	Karyotype	MLL-EP300 breakpoint	Latent period (months)	Primary malignancy	Cytotoxic exposure	Reference
1	4/M	AML	48,XY, +8, +8, t(11;22)(q23;q13)	MLLexon9/EP300exon15	67	NHL	Chemo including ETP	[16]

2	5/F	AML	46,XX, t(1;22;11)(q44;q13;q23), t(10;17)(q22;q21)	MLLexon7/EP300exon15	36	Neuroblastoma	Chemo including THP,	[17]

3	66/M	AML	46,XY, t(11;22)(q23;q13). idem, +8	MLLexon10, 11/EP300exon13	16	PTCL NOS, AML with MDS	CBDCA, CPA CHOP-14, ESHAP	[18]

4	62/F	CMML	46,XX, t(11;22)(q23;q13)	MLLexon10/EP300exon15	10	ATL	mLSG+ mogamulizumab	Present case

CHOP-14 contains cyclophosphamide, doxorubicin, vincristine, and prednisolone. ESHAP consists of etoposide, methylprednisolone, cytarabine, and cisplatin. ETP, etoposide; THP, pirarubicin; CBDCA, carboplatin; CPA, cyclophosphamide. mLSG contains VCAP (vincristine, cyclophosphamide, doxorubicin, and prednisone), AMP (doxorubicin, ranimustine, and prednisone), and VECP (vindesine, etoposide, carboplatin, and prednisone). NHL, non-Hodgkin lymphoma; PTCL NOS, peripheral T-cell lymphoma not otherwise specified; ATL, adult T-cell leukemia/lymphoma.
